# Wicking pumps for microfluidics

**DOI:** 10.1063/5.0218030

**Published:** 2024-11-01

**Authors:** Behrouz Aghajanloo, Wil Losereewanich, Christopher J. Pastras, David W. Inglis

**Affiliations:** School of Engineering, Macquarie University, Sydney, Australia

## Abstract

This review describes mechanisms for pulling fluids through microfluidic devices using hydrophilic structures at the downstream end of the device. These pumps enable microfluidic devices to get out of the lab and become point-of-care devices that can be used without external pumps. We briefly summarize prior related reviews on capillary, pumpless, and passively driven microfluidics then provide insights into the fundamental physics of wicking pumps. No prior reviews have focused on wicking pumps for microfluidics. Recent progress is divided into four categories: porous material pumps, hydrogel pumps, and 2.5D- and 3D-microfabricated pumps. We conclude with a discussion of challenges and opportunities in the field, which include achieving constant flow rate, priming issues, and integration of pumps with devices.

## I. INTRODUCTION

Microfluidics, known for its proficiency in handling micro- and nano-liter volumes of fluids, has facilitated revolutionary progress across biomedical and biochemical applications. Yet the potential of the work is often stuck in laboratories, where a typical device is more a “chip-in-a-lab” than a “lab-on-a-chip” (LOC). This is because accessories such as fluid pumps and analytical instruments are large, external, and expensive.^1,2^ By integrating the pump into the device, microfluidics has great potential for translational impacts in healthcare, environmental monitoring, and other fields.^3–5^

Pumpless microfluidics was established in the 1980s as a branch of microfluidics technology aiming for self-contained, easy-to-use, and cost- and time-effective devices.^6,7^ Early efforts including wettability-based, gravity-driven, lab-on-a-disk (centrifugal), and capillary-driven microfluidics demonstrated reliable and sophisticated flow control. In 2005, the pioneers of capillary microfluidics, Delamarche, Juncker, and Schmid, produced a review of their work in microfluidics.^8^ They described a capillary system as a device where fluid handling is encoded into the physical and chemical features of the chip. The work addressed challenges like microchannel sealing, liquid displacement, sequential delivery, surface patterning, and analyte detection in microliter samples.

Ten years later, Boyd-Moss *et al.* presented a review on self-contained microfluidics.^9^ It addressed the limitations of conventional microfluidics, particularly their dependence on bulky external equipment, and introduced self-contained microfluidics as a workable alternative. These systems are noted for their enhanced control of fluid flow in diagnostic assays and potential integration with smartphone technology. In 2018, Olanrewaju *et al.* (Juncker's group again) published an outstanding review that introduced the concept of capillaric circuits;^10^ a library of elements with specific liquid handling functions. The term “capillaric circuits” was introduced to describe this modular assembly of elements and to distinguish it from paper microfluidics, capillary electrophoresis, and physical glass capillaries.

Thereafter, a review by Berthier *et al.* targeted microfluidic capillary systems where at least one side of the fluid channel was open to the air.^11^ These approaches can be simpler to fabricate as they typically do not require lidding or porting, while also eliminating the problem of bubbles arising in the sample. However, open-channel systems must remain under negative pressure, and as such, if placed vertically, the hydrostatic pressure can dispel liquid from the capillary channels. This capillary system is also highly susceptible to evaporation. Wettability patterning for open-channel fluidics was well reviewed by Mahapatra *et al.*^12^ Further, Narayanamurthy *et al.* covered the evolution of passively driven microfluidics,^13^ describing their underlying mechanisms, potentials, and limitations. Moreover, they conducted a comprehensive survey of published papers and patents of the field to elaborate the importance of developing compact, portable, and simple-to-operate LOCs to meet market demands.

The field received another review in 2020 by Hassan *et al.* investigating the role of capillary microfluidics in Point-of-Care (POC) diagnostics when combined with smartphone detection.^14^ This review covered fundamentals of capillary fluid movement and emerging POC devices for studying antimicrobial resistance. It noted challenges around flow stability and stability of surface treatments and coatings. In 2021, Delamarche *et al.* reviewed the potential of capillary microfluidics in enhancing medication adherence.^15^ The review suggested low-cost POC devices to detect pharmaceutical ingredients from body fluids to verify adherence to medicinal prescriptions.

Lastly, Azizian *et al.* released a review on capillary microfluidics with a focus on 3D fabrication. The review explored recent advancements of capillary microfluidics in POC devices, considering surface chemistry and 3D manufacturing.^16^ Despite a focus on 3D printing, the review covered devices that resemble those made using conventional soft lithography and did not cover the novel wicking structures covered in Sec. VI.

Several recent investigations offered insights into the expanding microfluidics landscape,^17–19^ revealing the market is expected to grow from US$5.1 billion in 2018 to an estimated US$22.7 billion in 2026.^19^ Companies including 1Drop Diagnostics (https://www.1dropdx.com/), Sensoreal (https://www.sensoreal.com/), and Criticare Dx (https://www.criticaredx.ca/) have emerged and focused on capillary microfluidics. IBM (a large American technology company) has supported pioneering research in this field since at least 2002^20^ and has recently extended their capabilities with a publication in 2020.^21^

Because pumpless microfluidic methods can greatly simplify the user experience of a microfluidic device, they are crucial to commercial uses, but it is difficult to quantify its current or potential impact beyond simple lateral flow assays. Academic and industrial research is likely to continue to make heavy use of expensive lab equipment because this equipment is now readily available and it enables systematic investigation of parameters such as flow rate.

Previous reviews have focused on the capillary features of microfluidic devices and their applications. This review is focused on wicking pumps, which we define as features whose sole purpose is to move fluid in the chip. We present a brief explanation of capillary pump fundamentals and then examine progress of four different types of capillary pumps: porous materials, hydrogels, and 2.5D- and 3D-microfabricated pumps. We then consider challenges and opportunities in the field.

## II. FUNDAMENTALS OF CAPILLARY PUMPS

Capillary pumps are like batteries. They store energy in the form of a dry surface or material that can achieve a lower energy state through hydration. When molecules of the material in the pump are hydrated, energy is released, causing fluid to move through the connected microfluidic device. A good description of the physics of wetting can be found in Chapter 10 of Probstein's Physicochemical Hydrodynamics.^22^

A common way to quantify the performance of a capillary pump is by plotting the position of the wetted front (*x*) vs the square root of time (*t*). This typically produces a linear plot as described by the Washburn equation,^23,24^

$$x(t)=\sqrt{\frac{\sigma r\cos\theta }{2\mu }}\sqrt{t},$$ (1)

where σ is the surface tension, *r* is the capillary radius, θ is the contact angle, and μ is the viscosity. The fluid initially moves quickly but slows as the fluidic resistance grows. The viscosity of air is approximately 50 times lower than water (at 20 °C and standard pressure), so the resistance caused by movement of air out of the wick can be ignored. This analysis suggests that a large pore radius (*r*) is always desirable, as it increases the flow rate and reduces resistance. However, this ignores the pump pressure, which can be measured by observing the wicking of fluid upward (against gravity).

An attractive force between the capillary wall and the liquid creates a capillary pressure (*P*_cap_) in the fluid, which pulls the liquid up against gravity. Making a few approximations, we can show that liquid will rise above the background fluid level to a height (*H*) of $H=2\left(\frac{\sigma }{Dg}\right)\frac{\cos\theta }{r}$,^22^ where *D* is the density of the liquid and *g* is the local gravitational acceleration. This equation is Jurin's law.^25^

Clearly then, the pressure caused by the hydrophilic interaction in the capillary is 2σcos⁡θ/r. If we assume that the surface is quite hydrophilic and cos⁡θ≈1 (e.g., cos⁡θ≈0.95), we can simplify to

$$P_{cap}=DgH\approx 2\sigma /r.$$ (2)

In compliment to the previous measure of horizontal movement, this equation suggests that a small pore size produces a pump with high pressure but slow flow, whereas a large pore results in a pump with low pressure but high flow. The competition between these two effects can be clarified by considering a porous material as composed of a collection of cylindrical capillaries whose diameters are equal to the mean pore size.

Each of the capillaries has a resistance *R* given by the Hagen–Poiseuille equation,

$$R=\frac{\Delta P}{Q}=\frac{8\mu L}{\pi r^{4}},$$ (3)

where *μ* is the fluid viscosity and *L* is the length of capillary. Those familiar with linear circuit theory will appreciate that the resistance of a homogenous object is given by $R=\frac{\rho L}{A}$, where ρ is the resistivity of the material and *A* is the area normal to the transport direction. This equation can be applied to the movement of liquid if the material is comprised of many small capillary channels.

Since the wetted length of a wicking pump changes during use, so does its resistance. It is, therefore, more useful to define the resistivity of the pump. Equating the electrical circuit theory equation for resistance to Eq. (3) and solving for resistivity (ρ) gives

$$\rho =\left(\frac{8\mu L}{\pi r^{4}}\right)\frac{A}{L}.$$ (4)

Any porous material comprises both solid and fluid regions, so the cross-sectional area is not just the pore area. A solid material has a porosity *m*, defined as the pore area divided by the total cross-sectional area. Therefore, the total area of the porous material is $A=(\pi r^{2})/m$. Placing this into Eq. (4) gives an equation for the resistivity of a porous material,

$$\rho =\frac{8\mu }{mr^{2}}.$$ (5)

The Washburn equation [Eq. (1)] can be re-cast in terms of capillary pressure and internal resistance by substituting Eqs. (2) and (5) into (1). Equation (6) explicitly shows how capillary pressure, internal resistivity, and porosity affect the flow rate in a porous material,

$$x(t)\approx \sqrt{\frac{2}{m}\frac{P_{\mathrm{cap}}}{\rho }}\sqrt{t}.$$ (6)

When a capillary pump is connected to a microfluidic device with significant fluidic resistance, the internal pump resistance is less important, and the pump pressure is most critical. Here, it would be wise to use a smaller pore size. Conversely, when connected to a device with low internal resistance, the internal resistance of the pump must also be low, and the pump pressure is less critical. Here, it would be wise to use a larger pore size. There are clear parallels with maximum power transfer theory in electrical circuits.

A collection of capillary tubes is a reasonable approximation of rigid porous materials. But, the approximation does do not suit materials that swell, such as hydrogels. Hydrogels lack a clearly defined wetting front or pore size, and their internal fluidic resistivity decreases with swelling.^26^ As the material hydrates and volume increases, the suction pressure that it can produce decreases. These features mean hydrogel suction pumps have very different fundamentals, but just like rigid porous membrane wicks, the flow speed decreases as the fluid is pumped.

After capillary pressure and internal resistance, the final parameter needed to describe the capillary pump is its capacity, which is the volume of liquid that it can intake. The volume is dependent on geometry, but a more fundamental property is the mass or volume efficiency. This is the mass or volume of the absorbed liquid divided by the final mass or volume of the wetted pump. For a rigid porous material, this is the porosity *m*. Hydrogels are often characterized by their swelling ratio, which is the volume of the hydrated material divided by the volume of the dry material. The swelling ratio can exceed 200:1, for a volume efficiency of 199/200 = 99.5%, but is affected by the salinity of the pumped fluid.^27^

## III. POROUS MATERIAL PUMPS

**FIG. 1.**
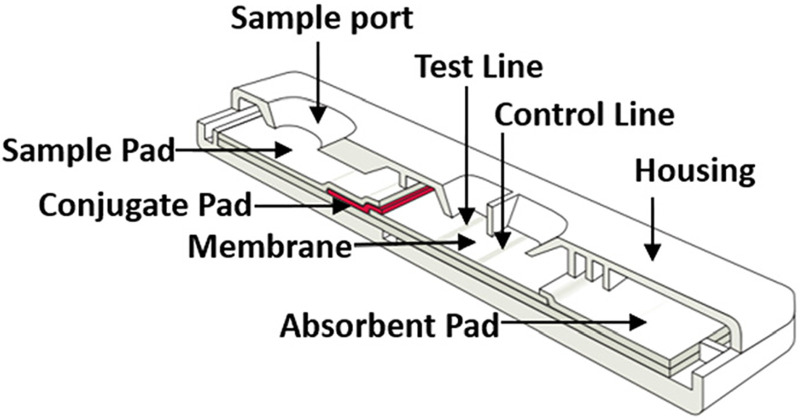
Schematic of a typical lateral flow assay showing four capillary fluidic elements: sample pad, conjugate pad, membrane, and absorbent pad. Image reproduced with permission from Lepowsky *et al.*, Biomicrofluidics **11**(5), 051501 (2017). Copyright 2017 AIP Publishing LLC.^28^

The oldest form of a microfluidic capillary pump is the traditional lateral flow assay.^28^ Lateral flow devices which rely on chromatography papers date back to 1960.^29^ Figure 1 shows the composition of a modern lateral flow strip comprising four separate porous material pieces: a sample pad, a conjugate pad containing dry reagents, a membrane where analytes are bound, and an absorption pad to draw adequate liquid through the membrane. Each piece acts as a pump, but the dominant pump (in terms of capacity) is the absorbent pad. There has been considerable work done in the microfluidics community on paper-based devices, which, like lateral flow assays, integrate the wicking action throughout the device. These two large fields and are not covered here.

**FIG. 2.**
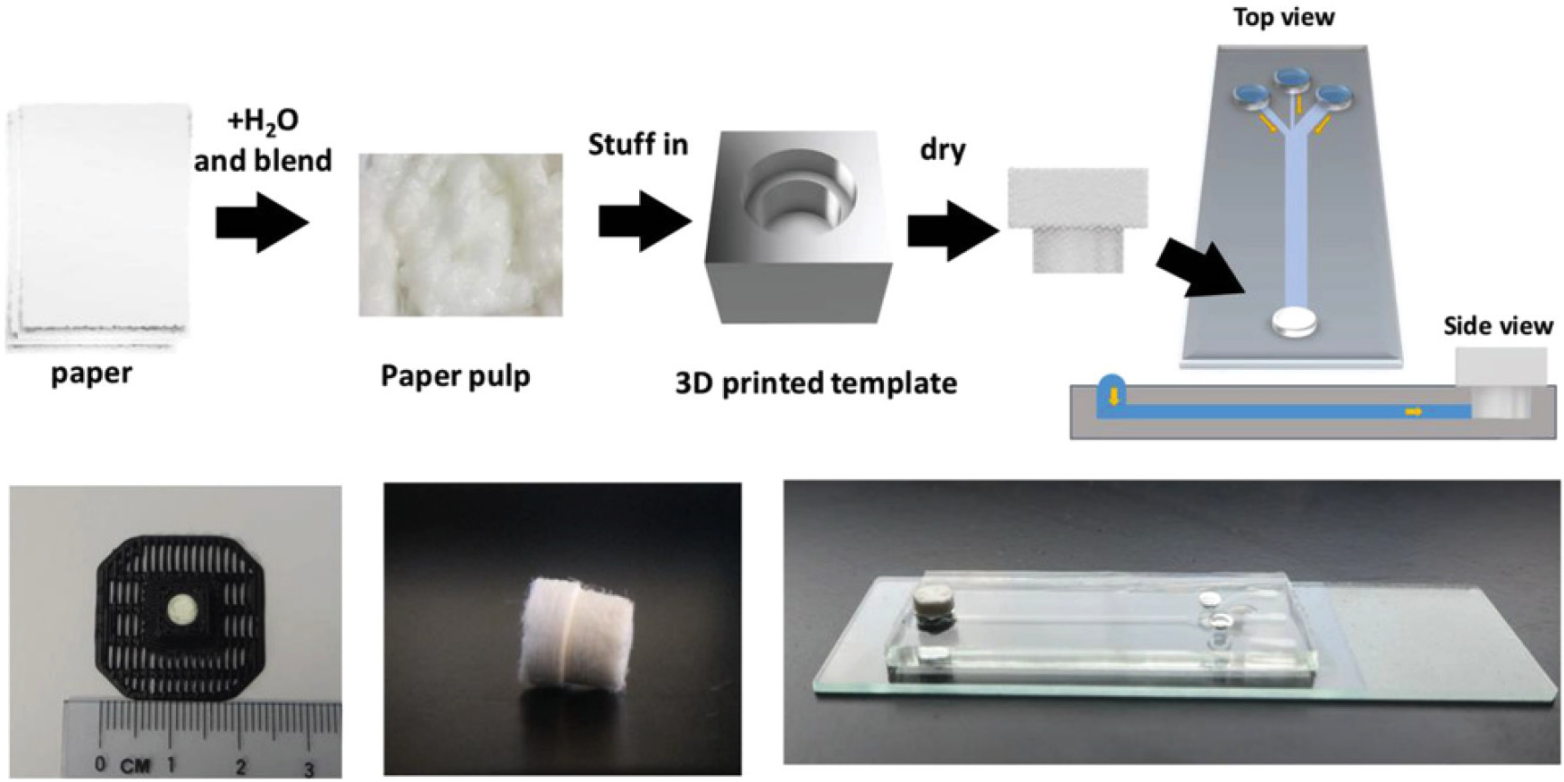
Fabrication and use of a pulped paper plug as a capillary pump for a particle separation device. Image reproduced with permission from Salafi *et al.*, Adv. Mater. Technol. **4**3, 1800359 (2019). Copyright 2019 Author(s), licensed under a Creative Commons Attribution (CC BY) license.^31^

The most common configuration for a capillary pump is a porous material at the exit or end of a microfabricated device. In 2004, Wolf *et al.* used centimeter-scale clean room paper pieces to wick sample through a microfabricated device for the detection of cardiac biomarkers, such as C-reactive protein, myoglobin, and cardiac Troponin 1.^30^ The pieces were cut using a plotter cutter and inserted into a milled plastic device. In 2019, Salafi *et al.* molded pulped paper into a custom-shaped wicking-plug for particle separation [Fig. 2].^31^ More recently, Tokihiro *et al.* studied the fluid dynamics of various fluids in an open-channel microfluidic device with integrated Whatman #1 filter paper as pumps.^32^

The pumping properties of porous material pumps such as filter papers have been quantified in a few papers. Wang, Hagen, and Papautsky compared the ability of Whatman #1 (11 *μ*m pores) and #4 (25 *μ*m pores) papers to pump liquid through a simple device.^33^ They found #4 (25 *μ*m pore) had a higher flow rate, but did not quantify capillary pressure, internal resistance, or the microfluidic device's resistance. Kokalj *et al.* used Schleicher and Schuell Grade 595 paper. They claimed 3800 Pa of capillary pressure, but did not give details of how this relatively high number was measured.^34^ Delon *et al.* used a woven fabric-based pump for organ-on-a-chip work and found a capillary pressure of about 700 Pa (70 mm H_2_O).^35^ Recently, we measured the capillary pressure of Whatman #1 (11 *μ*m pore) with water to be 2100 Pa and found an internal resistivity of 2.8 Pa s/mm.^2^^36^

Filter paper is a very popular pumping material, but this is most likely the result of convenience rather than deliberate engineering. Filter papers are readily available in various pore sizes, which is helpful because it allows users to tune the pressure and internal resistance to match the microfluidic device. But filter paper is designed to withstand more force than is needed in most microfluidic wicks. This strength necessitates low porosity and low volume efficiency. Non-paper porous materials such as absorbent pads have higher porosity and, therefore, can have higher capacity and lower internal resistance, while achieving similar capillary pressure. For example, Parandakh *et al.* and Yafia *et al.* used glass fiber conjugate pads and electrophoresis blotting paper supported by filter paper as high volume wicks.^37,38^

Finally, when considering pore size, it should be larger than any particulates in the solution being pumped. Accumulation of particles at the entrance to the pump may drastically increase internal resistance and reduce flow rate.

## IV. HYDROGEL PUMPS

**FIG. 3.**
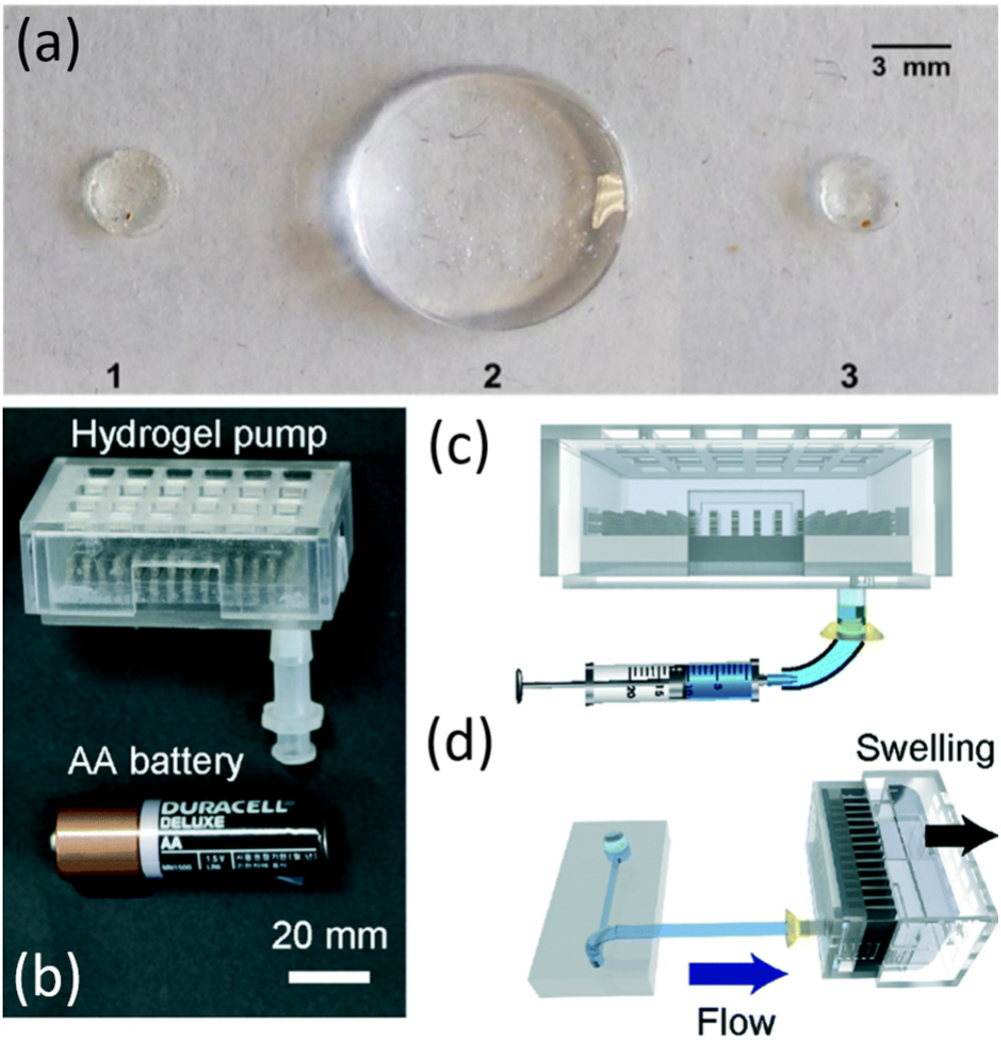
(a) A hydrogel drop before swelling (1), after soaking in water for 2h (2), and following drying at room temperature (3). Image reproduced with permission from Akyazi *et al.*, Sens. Actuators B Chem. **261**, 372–378 (2018). Copyright 2018 Author(s), licensed under a Creative Commons Attribution (CC BY) license.^40^ (b) Photograph of the hydrogel-driven microfluidic suction pump, with an AA battery for size comparison. (c) Schematic of water injection into the hydrogel pump. (d) Hydrogel swelling as suction pump removes water. Images reproduced with permission from Seo *et al.*, Lab Chip **19**, 10 (2019). Copyright 2019 Royal Society of Chemistry.^42^

Hydrogel pump materials are weakly bonded polymers with a high and homogenous water content. As water is absorbed by the polymer, bonds break and re-form, allowing the hydrated material to swell. While hydrogels can act as very effective absorbers of water, their first use as a pump in microfluidics may have been used as a reservoir. Niedl and Beta stored water in a hydrogel on chip which was released upon an increase in temperature for a positive pressure pump.^39^ In 2018, Akyazi *et al.* added a small drop of dehydrated hydrogel to the outlet reservoir of a paper-based device.^40^ This droplet increased the transport capacity of the paper device by nearly tenfold [Fig. 3(a)]. Shay *et al.* used a hydrogel midway through a device^41^ and claimed that it caused transport both into and out of the hydrogel chamber, but the described mechanism is not convincing.

The ability of a hydrogel to absorb large amounts of water was demonstrated by Seo *et al.*^42^ They placed sodium acrylate particles around a rigid support structure inside an expandable chamber. With this, they absorbed over 20 ml of water with pressures dropping from 3000 to 500 Pa. The pump is shown in Figs. 3(b)–3(d). Compared with rigid porous material pumps, the mass or volume efficiency of hydrogel pumps can be much higher, but the capillary pressure appears lower and is reduced further if the pumped fluid is salty.^27^

## V. 2.5D MICROFABRICATED PUMPS

**FIG. 4.**
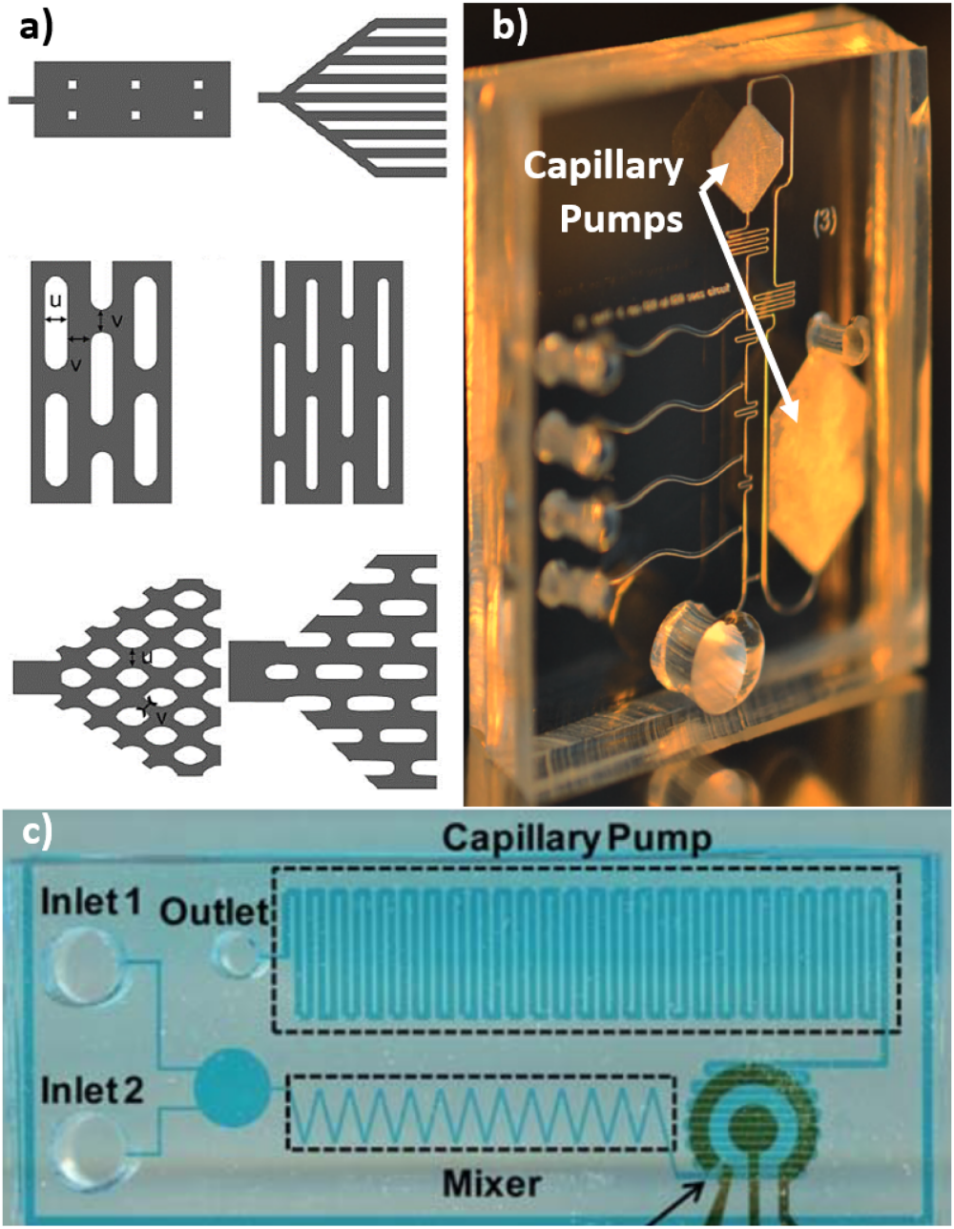
Microfabricated capillary pumps. (a) A selection of designs tested by Zimmerman *et al.* Image reproduced with permission from Zimmermann *et al.*, Lab Chip **7**, 1 (2007). Copyright 2007 Royal Society of Chemistry.^44^ (b) The device used by Safavieh and Juncker contains two obstacle arrays as integrated pumps. Image reproduced with permission from Safavieh and Juncker, Lab Chip **13**, 21 (2013). Copyright 2013 Royal Society of Chemistry.^45^ (c) A capillary pump made of a serpentine channel. Image reproduced with permission from Lillehoj *et al.*, Lab Chip **10**, 17 (2010). Copyright 2010 Royal Society of Chemistry.^43^

The simplest microfabricated pump is just a hydrophilic channel.^43^ A more robust approach that is less susceptible to clogging is an array of hydrophilic obstacles. The most significant seminal work on this topic was by Zimmerman *et al.*,^44^ though similar structures were described in 2005 by Delamarche *et al.*^8^ These pumps are typically a large region within a microfabricated chip that is full of high-density periodic obstacles with vertical sidewalls. We call them 2.5D because they are two-dimensional patterns that are extruded straight between the channel floor and ceiling. The surfaces must be hydrophilic, but often the sealing surface has a different contact angle than the other three sides. Figure 4(a) shows examples of microfabricated capillary pumps tested by Zimmerman *et al.*^44^ The various shapes highlight the effect of pinning, where the advancing liquid front prefers to stay at sharp corners. The shape and arrangement of obstacles can create anisotropic movement of the wetting front. Figure 4(b) shows an example of two microfabricated 2.5D pumps integrated into a device.^45^

The pumps do not need to be complex obstacle arrays.^43,46^ Figure 4(c) shows a pump that is just a hydrophilic serpentine channel. Although the shapes differ, the fundamental concepts of contact angle, pore size, and porosity still apply. Hence, a high-performance pump is very hydrophilic, has a high density of very small features, and has a pore size that is well suited to the pressure, flow requirements, and particulates of the application. It is easier to integrate these pumps into devices than it is to integrate a porous material like paper; however, paper inserts typically have higher volume capacity and can achieve high pressure without high resolution lithography. 2.5D pumps come at the expense of chip “real-estate,” which can be costly in production setting. There has not been much development of these types of pumps recently; instead, more focus has been placed on modulating geometry and surface features in chips to create complex autonomous fluid control.^45^

## VI. 3D-MICROFABRICATED PUMPS

**FIG. 5.**
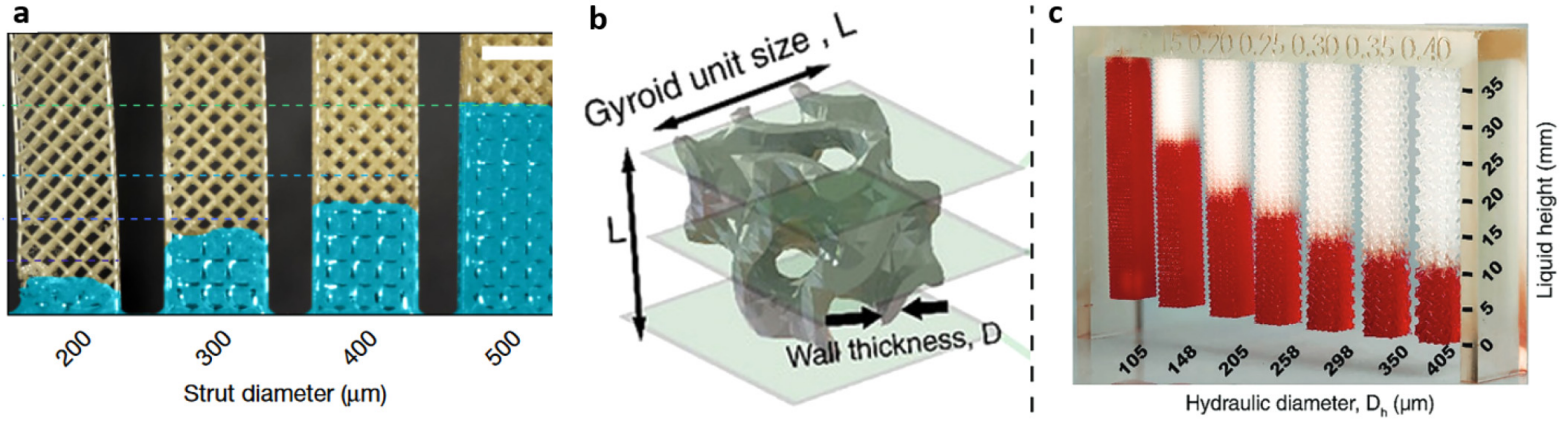
Periodic 3D wicking structures. (a) Vertical columns of the body centered cubic unit cells with false-colored liquid showing an increasing capillary pressure with larger strut diameter and smaller pores. Image reproduced with permission from Dudukovic *et al.*, Nature **595**, 7865 (2021). Copyright 2021 Springer Nature.^48^ (b) The gyroid unit cell used by Karamzadeh *et al.* and (c) red colored water is wicked higher in 3D-printed columns with smaller unit cells. Images reproduced with permission from Karamzadeh *et al.*, Adv. Mater. **35**(47), 2303867 (2023). Copyright 2023 Author(s), licensed under a Creative Commons Attribution (CC BY) license.^49^

3D printing has allowed the creation of novel wicking structures with the potential for integration with high volume capacity. 3D-printed structures for wicking in heat pumps likely predated its use in bio-microfluidics.^47^ Very recently, Dudokovic *et al.* created porous structures with controlled pore sizes and wicking paths.^48^ They used periodic arrays of cubic unit cells that are similar to that used by Jafari *et al.*^47^ They showed water rise of about 22 mm (220 Pa) [Fig. 5(a)].

Karamzadeh *et al.* created triply periodic minimal surface unit cells with feature sizes that can be scaled to deliver different capillary pressures [Figs. 5(b) and 5(c)].^49^ The unit cells have high surface area to volume ratios, low flow resistance, and consistent wall thickness. These characteristics deliver better wicking properties than the body centered cubit unit cells used earlier. The authors used a modified 3D-printing resin that showed an improved hydrophilic contact angle of ∼35°. A unit cell of 700 *μ*m and a hydraulic diameter of 100 *μ*m achieved a water height of at least 35 mm (350 Pa). To date, this is the smallest unit cell that has been printed as a wick. The parameters suggest a capillary pressure of about 2300 Pa or a wicking height of about 240 mm. 3D-printed wicks are unlikely to match the capillary pressure or low cost of amorphous materials like paper; however, designers can modulate the properties throughout the wick control flow rate.

3D printing of all kinds of microfluidic devices is rapidly developing as improvements are made to printing technology, with higher resolution, lower cost, and shorter print times. These changes will mean more 3D printing and less photolithography. Despite this trend, further work is needed to improve water contact angles of 3D-printed materials.

## VII. CHALLENGES AND OPPORTUNITIES

The physics of fluid movement within pumpless microfluidic chips is governed by a complex interplay of forces at the microscale, particularly surface tension, viscous forces, and attractive forces between liquids and surfaces. These factors directly influence the pressure and flow rate of capillary pumps.

Capillary pumps typically produce monotonically decreasing flow rates. This is because the capillary pressure is usually constant, while the internal fluidic resistance of the wetted system increases as liquid displaces air. For a swelling material like a hydrogel, we expect the capillary pressure to drop faster than resistivity as the material swells, resulting in a reduced flow rate. A monotonically decreasing flow rate is usually undesirable; Guo *et al.* had considered the problem and achieved a practically constant flow rate by integrating a very high constant fluidic resistance element in the pump that dominated all other variable resistances.^50^ This only solves the problem by drastically reducing the flow rate. Increasing the volume in the pump as the wetting front progresses reduces the effect but does not eliminate it. See Fig. 5 of Azizian *et al.* for an example.^51^

It is possible to flatten the flow rate by changing the wick's pore size or contact angle as the wetted front moves through it. An analytical solution for microfabricated pumps was presented by van der Wijngaart.^52^ This would make an interesting project to demonstrate in a 2.5D- or 3D-microfabricated pump but does not appear to have been demonstrated yet. Li *et al.* achieved constant flow rate in a pump by integrating a heater to reduce the contact angle of the wetting front, thereby pulling harder as the pump filled up.^53^

Microfabricated pumps with low contact angles have a tendency to entrap bubbles. These trapped bubbles increase resistance and reduce fluid movement, thereby reducing the capacity of the pump. Safavieh showed 2.5D microfabricated designs that prime without trapping bubbles.^54^ Priming of the microfluidic device (not the pump) is possible but can be challenging because the user must stop the fluid before the pump.^36^ Some devices will self-prime reliably; for designs that will not, they must be wetted and degassed prior to being connected to the pump.

It is not practical to fabricate the pump on the same substrate as the chip as the feature sizes and surface treatments may be different. This means that a pump must be connected to the chip. There is no standard for how microfluidic devices connect to tubing, and the field is even further from consensus on how a wicking pump would connect. The recent work by Parandakh *et al.* to connect a large porous pump to a complex chip appears to have taken inspiration from integrated electronics and is innovative and elegant.^37^

## VIII. CONCLUSION

In this review, we have considered capillary pumps as discrete elements that drive fluid flow in a microfluidic device. Such pumps have the potential to facilitate the translation of microfluidic research from lab-based demonstrations to user friendly, low cost, point-of-care systems, or subsystems. The idea of a capillary pump is older than the field of microfluidics, yet significant innovations from the combination of the two continue. We highlighted the importance of capillary pump pressure, internal resistance, and pump capacity in pump performance and how they may change depending on the device features. Outstanding challenges relate to the integration of pumps with devices, connectivity between pumps and devices, pump capacity, and achieving constant flow rates in devices connected to wicking pumps.

## Data Availability

Data sharing is not applicable to this article as no new data were created or analyzed in this study.
